# The Colonial Medical Service

**Published:** 1911-09-02

**Authors:** 


### THE COLONIAL MEDICAL SERVICE.

### WHAT IT OFFERS TO THE NEWLY-QUALIFIED MAN.

A large proportion of medical men on their first attaining the dignity of graduation are faced with the difficulty as to what branch of the medical profession they shall embrace. It is not open to every man to have a wealthy father who will maintain him in Harley Street until he is able to obtain a practice as a consultant, and only a small proportion have fathers in private practice who will accept their sons as partners and leave to them a family practice. The great majority must look further afield. The Services, with their attractions and demerits, are known to many, but it does not appear that the average man on finding himself equipped is aware of the large and interesting field that lies open to him in the Colonies and great Dependencies of the Empire.

### How to Enter the Service.

The Colonial Medical Service offers to younger men an opening of exceptional interest and the attainment in many instances of a high position in their profession, but instead of the Services being concentrated, as in the case of the Indian Medical Service, the Army, and Navy, employment under the Colonial Office is divided into many branches. Especially is this the case in the Crown Colonies which, though locally controlled by Legislative Councils, manage their affairs through the Government at home in very varying manner. Not only does the pay differ to a great extent, but the conditions of service differ still more. The periods for which men must serve to obtain a pension vary considerably, and there is always the question of permission, or not, to embark in private practice outside the official routine of the appointment.

At no distant date some of the African Colonies were administered under the Foreign Office, while but a little time ago the various Colonies on the West Coast of Africa selected their officers independently of one another. Of late years, however, a considerable change has taken place. Practically all the Crown Colonies and Protectorates are now under the Colonial Office, while the West African Colonies, though still administered by the Colonial Office, have been formed into a distinct Service under the title of the West African Medical Service, more familiarly known as the "Wams."

The majority of the Crown Colonies are within the tropics, so it is necessary to provide medical officers who are equipped with a knowledge of the diseases peculiar to hot climates. For this purpose candidates are appointed to most of the Colonies on the condition that they obtain the certificate of the London or Liverpool School of Tropical Medicine. To this end arrangements are made by which the Crown pays for tuition and three months' board and residence for each officer on appointment, together with an allowance of 5s. per diem while he is under tuition. These concessions, however, are made on the condition that the candidate enters into an agreement to refund the amount expended on his tuition, board and residence if he fails to obtain the certificate of the school—a matter of £48.

In giving an account of the Colonial Medical Service one must not overlook the indebtedness of the Colonies and the medical officers to Mr. Joseph Chamberlain. His untiring zeal in the interests of the tropical Colonies first made apparent the need for reform, and the changes made when he was Colonial Secretary were the initial steps which have brought the service to its present position of importance and influence.

### The West African Medical Staff.

The West African Medical Staff was established about twelve years ago, and reconstituted as a result of the report of the Departmental Committee appointed by Lord Crewe in 1909. It includes the following West African Colonies and Protectorates—namely, Southern and Northern Nigeria, the Gold Coast (including Ashanti and the Northern Territories), Lagos, Sierra Leone, and the Gambia, and at the present time the establishment just numbers two hundred medical officers. There are three principal medical officers, eleven deputy and provincial medical officers, and fifteen senior medical officers. Some of the above are detailed for sanitary duties, and form a separate class with additional remuneration. Selection for the staff is made by an advisory committee appointed by the Secretary of State. Candidates in the first instance are required to make application to the Private Secretary to the Secretary of State, Colonial Office, S.W., for their names to be entered on the register, and they will receive a form of application. A certain number of candidates are then invited to appear before the advisory committee, and on the recommendation of this committee appointments are made. Candidates are seldom selected who are over thirty years of age, and there being now a steady flow of men applying for the staff it is necessary to show good qualifications and to submit exceptionally good references. The pay on entering the service varies from £400 to £500 according to the particular Colony in the group to which the officer is appointed, and rises by annual increments of £20 from medical officer to senior medical officer at £600 or £700 per annum. The higher grades of deputy principal medical officer and principal medical officer advance by larger increments to £1,200 per annum in Southern Nigeria. Duty pay—that is, pay while actually performing his duties or for acting in a senior capacity or for extra work—frequently increases the senior officer's pay by £100 or more. Other additions include horse allowance, free transport of stores, and travelling allowances, and there are other additions where officers are engaged on military expeditions. Each officer on appointment receives £12 for outfit. Free furnished single quarters are provided at all recognised stations, and all medical requirements, including instruments, drugs, and microscope, are supplied by the Government. First-class passage out and home is provided, generally by the Elder-Dempster line sailing from Liverpool; the vessels are commodious and provide adequate and comfortable accommodation. Leave conditions are good, and practically amount to a year on the coast and six months on full pay away from it, leave being counted from the coast so as to give the officer four months clear in England.

The outfit is not costly, and no uniform has to be provided. The very moderate list of requirements can hardly be bought for the amount of the allowance, but much depends on individual needs. Officers on appointment are given a selected outfit list, and this can be added to to suit individual requirements. Many officers are able to augment their income by private practice, but the amount obtainable in professional fees differs considerably in different stations. An officer is only permitted to engage in private practice when this does not interfere with the discharge of his official duties, but with tact and discretion a good deal may be done in some places. Probably the average may be put down at £50 per annum, but in some of the large towns and on trade routes several hundreds a year may be added to the official salary. As regards pensions, an officer on attaining the age of fifty, or after having served for eighteen years, which includes the periods of leave on full pay, may retire on a pension calculated at the rate of one-fortieth of the last annual salary for each year of service, or if invalided at the same rate after seven years. A further regulation gives an officer the option to retire at the end of nine years' service on a gratuity of £1,000, or at the end of twelve years on a gratuity of £1,250.

The conditions of life on the West Coast of Africa have materially improved of late years. Arrangements have been made by which not only the medical staff but all officers are required to live outside the native towns and settlements. This has had a beneficial effect on the health of Europeans generally, and with the added knowledge of tropical medicine and hygiene now made available, many men return in no way materially affected by the climate. Some individuals appear to be immune to most forms of tropical disease, while others are able to maintain health by a strict regard to the altered requirements of diet and dress. The old curse of drink is dying out, and greater moderation in all things is being observed by the great majority of men.

The Secretary of State appoints to all the remaining Colonies and Protectorates that are administered through the Colonial Office, and applications for employment should be made to the Assistant Private Secdetary, Colonial Office, S.W. The age of candidates must be between twenty-three and thirty-five years of age, and they must be doubly qualified and registered. In nearly every case a free first-class passage out and home is given. This includes journeys home on leave. Pensions are granted at the usual rates that apply to other Government officials, and are based on the Superannuation Act of 1859, which gives roughly one-sixtieth of the maximum annual pay and emoluments for each year of service. Leave is usually granted, unless otherwise stated, at the rate of three months on full pay for every two years of service, and officers are in most Colonies permitted to accumulate their leave so as to enable them to spend six clear months in England. Medical officers are as a rule required to obtain a certificate that they have attended a course and obtained a certificate at a school of tropical medicine.

Medical officers for the Protectorates of Nyassaland, Uganda, Somaliland, and East Africa are in the first instance appointed as temporary medical officers, and are mostly engaged in the work in connection with sleeping sickness. As vacancies occur these temporary officers are absorbed into the service. Varying increments of pay accrue annually. The senior medical officers and Medical Officers of Health receive from £500 to £600, and the principal medical officers of health £750 to £850. Officers are allowed two months' leave on full pay every year, and are allowed to accumulate their leave.

The Federated Malay States and Straits Settlements regulate their medical services on practically the same lines, and these differ in some respects from other Colonies. On first appointment a medical officer becomes a house surgeon at a rate of pay varying from £300 to £400, usually with free quarters, and he may remain at this rank for about three years; as vacancies occur in the upper branches of the service these house surgeons are absorbed in Grade II. The pay then rises from £420 to £600, and in Grade I. from £600 to £700. Senior medical officers, £800 to £900; and principal medical officers, £1,200. House surgeons are not allowed private practice while serving as such, and in certain other grades private practice is restricted. A deduction of 4 per cent. is made from all salaries towards the Widows' and Orphans' Pension Fund.

In Hong Kong salaries vary from £480 to £700 in the junior ranks, to £800 to £1,000 for the principal civil medical officer. No private practice is allowed, and vacancies are almost always filled by promotion from another Colony. Four per cent. is deducted from salary for the pensions of widows and orphans.

Most of the appointments in Ceylon are filled by natives with British qualifications, with the exception of the Bacteriological Institute. The pay is good—£600 to £1,400. Salaries are paid in rupees, and consequently there is a considerable loss on exchange.

Mauritius offers a few good appointments, but the service is mostly recruited locally.

Fiji presents a good opening for a number of men. The pay commences at only £300, but it rises fairly rapidly to £500, and the chief medical officer has £800 a year. There is a house allowance of £50, and in some districts a medical officer is also a magistrate with an additional salary. Frequently the officer has charge of a district hospital. Some stations afford good private practice.

In Seychelles very similar conditions prevail, but on a smaller scale.

There are a few other scattered appointments in Gibraltar, Cyprus, St. Helena, and Falkland Islands at about the aforementioned rates of pay, but as these Colonies only support two or three medical officers there are not often vacancies.

The West Indies, including British Honduras, British Guiana, Jamaica, Trinidad, Windward and Leeward Islands, are on a different footing in some respects. British Guiana offers good openings after a period of probation. Candidates for this Colony must have been house physician or house surgeon in a general hospital, and are then appointed on two years' probation at £300, with quarters. The permanent staff is selected from these juniors. Medical officers receive pay at rates varying from £400 to £600, while the Medical Inspector receives £800, and the Surgeon-General £900. Private practice is reserved for the senior officers. All grades contribute 4 per cent. to the Widows' and Orphans' Fund. In the other West Indian Colonies the Government work is mostly done by district officers, which practically means that the appointments are held by general practitioners, some of whom are natives of the islands with British or American qualifications. The pay, which really amounts to a retention fee, is generally from £100 to £300 per annum.

In the Windward Islands, which include Grenada, St. Lucia, and St. Vincent, the pay rises in some instances to £400; and in British Honduras there are some appointments of this value where the medical officers act as District Commissioners at an increased rate of pay. In Trinidad also there are some appointments rising to £400. With the exception of British Guiana, the pay or honorarium in the West Indies does not carry a pension, though in most instances a deduction of 4 per cent. is made for widows and orphans.

Throughout the Colonial Service there are a considerable number of Medical Officers of Health usually called sanitary or health officers. They are required to hold a D.P.H., and the pay is usually about 25 per cent. above the ordinary rates.

A feature of the Colonial Service which has come much into notice during recent years is the establishment of research laboratories. The most important of these is the Institute of Medical Research, Federated Malay States, where the laboratories are spacious, and equipped with the latest requirements for modern research. There are also laboratories, though not quite so large, in British Guiana, Ceylon, and elsewhere. The directors of these laboratories are well paid, and the appointments are worth the consideration of any advanced research student.

Study leave on half-pay is now granted in most Colonies.

The demand for instruction in tropical medicine and hygiene has resulted not only in the establishment of tropical schools, but no less than three diplomas in tropical medicine have been instituted. Cambridge, owing to the indefatigable zeal of Professor Nuttall, was the first to invite candidates for a diploma in tropical medicine and hygiene (D.T.M. and H. Camb.). This diploma is open to all students of British nationality or holding registrable British qualifications. Then followed Liverpool, with its diploma in tropical medicine (D.T.M. Liverpool), open only to students of the Liverpool school and graduates of that University, and this year has been instituted the diploma in the diseases and hygiene of the tropics (D.T.M. Eng.) by the Conjoint Board. This diploma is open to all holders of a registrable qualification. The Cambridge examinations are held in January and August, and the London examinations will be held in April and October. Besides these, tropical medicine has been admitted as a sixth alternative subject for the M.D. of the University of London.

Probably 50 per cent. of the men who attend the tropical school have themselves returned from the tropics; they realise more than those who have not yet been abroad the difficulties of diagnosis and treatment of cases which they have never had an opportunity to study, and which are seldom seen in temperate climates. These men are sometimes very short of time, and can only just manage to put in a three months' course while on leave; to them it is of much importance that the examinations for the diplomas of Cambridge and London are held practically every three months

Life in the tropics has its drawbacks, and perhaps men do not find it as easy to get on there with their fellows as in more temperate climes, but, on the whole, those who succeed like the life, and generally appear to have a good time. Those who fail do not do so on professional grounds; it is rare to hear any complaint on this count, but, unhappily, there have been a good many failures on the medical side of tropical life. Too many men arrive in the Colony with the idea that they know better not only how to treat their patients and to look after the sanitary conditions, but also how to govern the Colony and to do everybody else's business better than the oldest resident. It is a remnant of the attitude of the average house surgeon, and is fatal to peace in a small community. To make a success of it, the newly-appointed medical officer must throw in his lot with those he joins—there must be much give-and-take, and the new man must realise that the Governor and the Colonial Secretary and the Officer of Police and the rest, all have their difficulties, which they would like to adjust if they could. The medical officer sees some alteration or sanitary improvement obviously necessary, some change in rules that his recent touch with the latest developments at home tell him are necessary, and because these matters are not attended to at once he cuts up rough. It takes some time for him to realise that administrators and others are doing their best, often at considerable disadvantage, far distant from the controlling power at home. Meanwhile, to use a vulgar expression, the fat is in the fire, and the man who went out full of confidence has to come home. The newly-appointed officer must sink all preconceived ideas of what should and what should not be in a tropical colony, throw in his lot cheerfully with those with whom he has to live, and in course of time he will find that others besides himself are trying to do their level best. In this frame of mind he will come back on leave with the idea that the Colonial Service is not so bad after all, and that there are many good fellows in it.

